# Integrated network pharmacology and metabolomics reveal the mechanisms of *Jasminum elongatum* in anti-ulcerative colitis

**DOI:** 10.1038/s41598-023-49792-w

**Published:** 2023-12-17

**Authors:** Jinyan Qiu, Guanlin Xiao, Minjuan Yang, Xuejun Huang, Dake Cai, Canhui Xie, Zhao Chen, Xiaoli Bi, Aili Xu

**Affiliations:** 1 School of the Fifth Clinical Medicine, Guangzhou University of Chinese Medicine, Guangzhou, 510405 China; 2 Guangdong Province Engineering Technology Research Institute of Traditional Chinese Medicine, Guangdong Provincial Key Laboratory of Research and Development in Traditional Chinese Medicine, Guangzhou, 510095 China

**Keywords:** Drug discovery, Diseases, Gastroenterology

## Abstract

*Jasminum elongatum* (JE), an ethnic Chinese medicine, is widely used in the Lingnan region of China, because of its analgesic and antidiarrheal action, as well as its anti-inflammatory effects in gastrointestinal diseases. However, whether JE could against ulcerative colitis (UC) remains unclear. This research aims to reveal JE in treating UC and clarify the underlying mechanism. We used the 2.5% dextran sulfate sodium (DSS)-induced UC mice (C57BL/6J) to evaluate the therapeutic effects of JE. Metabolomics of serum and network pharmacology were combined to draw target-metabolite pathways. Apart from that, the targets of associated pathways were confirmed, and the mechanism of action was made clear, using immunohistochemistry. The pharmacodynamic results, including disease activity index (DAI), histological evaluation, and inflammatory cytokines in colon tissues, demonstrated that JE significantly relieved the physiological and pathological symptoms of UC. Network pharmacology analysis indicated 25 core targets, such as TNF, IL-6, PTGS2 and RELA, and four key pathways, including the NF-κB signaling pathway and arachidonic acid metabolism pathway, which were the key connections between JE and UC. Metabolomics analysis identified 45 endogenous differential metabolites and 9 metabolic pathways by enrichment, with the arachidonic acid metabolism pathway being the main metabolism pathway, consistent with the prediction of network pharmacology. IκB, p65 and COX-2 were identified as key targets and this study demonstrated for the first time that JE reverses 2.5% DSS-induced UC in mice via the IκB/p65/COX-2/arachidonic acid pathway. This study reveals the complex mechanisms underlying the therapeutic effects of JE on UC and provides a new approach to identifying the underlying mechanisms of the pharmacological action of Chinese natural medicines such as JE.

## Introduction

Ulcerative colitis (UC) is a nonspecific inflammatory bowel disease that is not only a difficult disease of the digestive system but is also associated with precancerous lesions of colon cancer^1–3^. UC is classified as a modern refractory disease by the WHO^4^. Moreover, the pathogenesis of UC remains unclear, and the effectiveness of pharmacological treatment for UC is limited. Currently, the primary pharmacological treatments for UC include 5-aminosalicylates, antibiotics, immunomodulators and biological agents. However, Western drugs are expensive, prone to relapse, and may have adverse effects, such as drug resistance. Therefore, it is essential to find new drugs that are inexpensive, safe and efficient.

As the mainstream of complementary and alternative medicine, traditional Chinese medicine (TCM) shows potential advantages with its safety and effectiveness, low cost, and few adverse effects. The multicomponent and multitarget characteristics of TCM make it feasible for the prevention and treatment of difficult diseases such as UC^5^. The main symptoms of UC are recurrent abdominal pain, diarrhea, mucus-purulent stools and other symptoms. TCM mainly treats UC with the use of herbs that clear heat and detoxify and resolve dampness the basis of evidence-based treatment.

*Jasminum elongatum* (Bergius) Willd. (JE), also called *Jasminum amplexicaule* Buch.-Ham. (Oleaceae) is a common Chinese herbal medicine from the Lingnan region of China and is recorded in *Standards of Chinese Herbal Medicines in Guangdong Province*^6^. According to a previous report, JE has the effect of clearing heat and detoxifying, resolving dampness and eliminating stagnation, and possesses significant anti-inflammatory and analgesic efficacy^7,8^. JE is used in China to treat gastrointestinal diseases clinically, such as abdominal pain and diarrhea^9,10^. In addition, ethanol extracts of JE can obviously inhibit TNBS-induced IBD, including UC symptoms in rats^11–13^. Although JE was analyzed as a possible effective herbal medicine for the treatment of UC, the mechanism of action of this pharmacodynamic substance has not yet been elucidated. Therefore, it is crucial to study its mechanism of action for the treatment of UC.

IL-1β is one of the pro-inflammatory factors that causes UC and can promote inflammatory cells to the site of inflammation and activate lymphocytes, among other effects^14^. Studies have shown that IL-1β levels are elevated in the colon of patients with UC during the active phase of ulceration, which suggests that IL-1β can be used as a criterion for the differentiation and diagnosis of UC^15^. Currently, IL-1β has been used as an important clinical indicator to determine the degree and efficacy of UC. TNF-α is a typical proinflammatory factor that can alter intestinal mucosal permeability and cause intestinal damage. Ngo, B showed that TNF-α is a key early signal in the inflammatory cascade. TNF-α levels are elevated in the blood, epithelial tissue, and feces of patients with active IBD^16^. TNF-α blockers, may exert a therapeutic effect on UC by blocking the binding of TNF-α receptors^17^. A literature study found that DAI, colon length and colon pathology section analysis of UC mice and colonic tissue inflammatory factor (IL-1β, IL-6, IL-10 and TNF-α) levels expression were significantly different compared to the control group^18–20^. A recent study has shown that JE can inhibit the level of pro-inflammatory factors (IL-1β, IL-6 and TNF-a) in IBD rat models^12^. Nevertheless, whether JE treatment of UC is related to pro-inflammatory factors needs to be further investigated.

**Figure 1:**
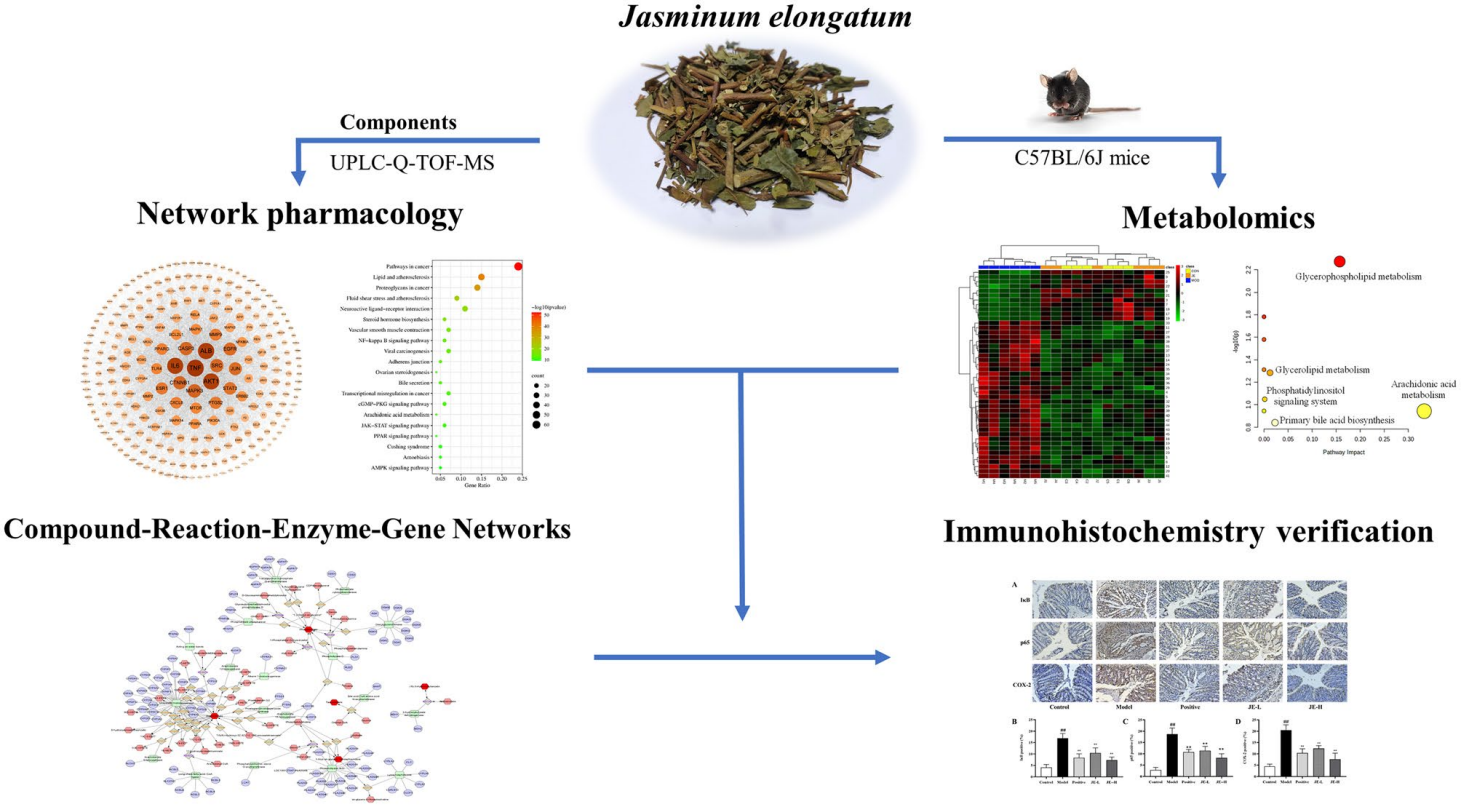
Schematic diagram of the research for UC treated with JE.

Metabolomics is omics research, that can be used to study the changes in endogenous metabolites in the body during treatment using traditional Chinese medicine^21–23^. The metabolome is located downstream of the gene regulation network and protein interaction network, and metabolomics studies are only conducted for potential metabolites and their related metabolic pathways, and thus lacking further exploration of their direct relationships. In contrast, network pharmacology is a novel research method focusing on upstream based on database mining and bioinformatics theory, which can link components, targets and pathways^24–28^. Network pharmacology is conducive to the in-depth study of pharmacodynamically active ingredients and mechanisms of action in traditional Chinese medicine. Therefore, the integration of metabolomics and network pharmacology for mechanistic studies allows them to complement and validate each other. This approach has emerged as a promising tool for investigating drug therapy for diseases in recent years^18,29–31^. These two research methods are similar to the "holistic concept" in TCM. A schematic diagram of the research is shown in Fig. 1. This study provides a direction for the mechanism of action of herbal medicine in the treatment of UC.

## Materials and methods

### Chemicals and reagents

*Jasminum elongatum* (Bergius) Willd. (JE, batch. no. N0720912) was provided by Guangdong Medicinal Materials Co., Ltd (Guangzhou, China). Voucher specimen No. N0720912 was deposited at Guangdong Province Engineering Technology Research Institute of Traditional Chinese Medicine, Guangzhou, China. Mesalazine enteric-coated tablets (5-aminosalicylic acid) were purchased from Losan Pharma GmbH. Dextran sulfate sodium salt (DSS) was obtained from mpbio. Occult blood test kits were obtained from Nanjing Jiancheng Bioengineering Institute (Nanjing, China). ELISA kits for IL-1β, IL-6, IL-10 and TNF-α were purchased from Tianjin Annuo Ruikang Biological Technology Co., Ltd (Tianjin, China). A hematoxylin–eosin (H&E) staining kit was purchased from Beijing Solarbio Science & Technology Co., Ltd (Beijing, China). Immunohistochemical reagents for IκB, p65 and COX-2 were obtained from Wuhan Servicebio Technology Co., Ltd (Hubei, China).

#### Animal and ethics statements

All experimental procedures were compiled with the NIH recommendations for the use and care of animals. The animal experimental protocols (No. 048937) were reviewed and approved by the animal ethics committee of Guangdong Provincial Engineering Technology Institute of Traditional Chinese Medicine (Guangzhou, China), and performed according to the relevant guidelines and regulations, also all methods are reported in accordance with ARRIVE guidelines. Thirty male C57BL/6J mice weighing between 18 and 22 g were obtained from the Guangdong Medical Laboratory Animal Center (Guangzhou, China). All the mice were fed a normal diet and housed in a barrier system at standard room temperature and 12 h light–dark cycle conditions. To verify that JE has an effect on UC mice, we divided mice into five groups: control (untreated), model (2.5% DSS), positive (2.5% DSS + 200 mg/kg mesalazine), JE-L (2.5% DSS + 7.8 g/kg JE) and JE-H (2.5% DSS + 15.6 g/kg JE)^8^. Except for the control group, all the mice in the other groups were treated with 2.5% DSS in drinking water for 5 days. The mice were treated for one week. Mental status, body hair color, gloss, weight change, fresh fecal viscosity, and fecal occult blood were observed daily. During the experiment, the percentage of daily weight loss, stool viscosity, and presence bloodstain condition of the mice in each group were recorded to calculate the disease activity index (DAI). The scoring criteria of DAI are displayed in Table 1.

**Table 1:** The DAI scoring rubric.

Body weight loss (%)	Stool viscosity	Presence bloodstain	Score
< 1	Normal	Negative	0
1–5	Soft stool	Light blue	1
5–10	Loose stool	Blue	2
10–20	Mucous stool	Dark blue	3
> 20	Mucous watery stool	Bleeding obvious	4

After 7 days of treatment, blood samples were collected from the eyeballs. Serum, colon and fresh fecal samples were collected from the mice for biochemistry assessments and metabolomics analysis. All samples were stored in a refrigerator at -80 °C for subsequent research.

#### Biochemical parameters and histopathology analysis

The colon tissues were measured colon length and used to estimate IL-1β, IL-6, IL-10 and TNF-α using mouse-specific ELISA kits. The colon sections were stained with hematoxylin and eosin, and then observed and photographed under a visible light microscope.

### Identification of chemical constituents in JE

#### Chemicals, reagents and materials

Methanol, formic acid, and acetonitrile of MS grade were purchased from Thermo Fisher Scientific (United States), and laboratory-deionized water generated by a Milli-Q system (United States) was used in all experiments. JE (batch. no. N0720912) was provided by Guangdong Medicinal Materials Co., Ltd (Guangzhou, China). Reference substances including protocatechuic acid, salicylic acid, caffeic acid, ethyl caffeate, rutin and scopoletin were all purchased from National Institutes for Food and Drug Control (Beijing, China). Chlorogenic acid was purchased from Chengdu Master Biotechnology Co., Ltd (Chengdu, China).

#### Drugs and reagents

The JE samples were prepared as follows: The JE was crushed and passed through a 355 ± 13 µm sieve. Then, we dissolved 2.0 g of powder in 25 mL of 50% methanol and ultrasonicated it (frequency of 40 kHz; power of 300 W) for 30 min. After cooling, shaking, and filtering, we took 5 mL of additional filtrate into a 10 mL volumetric flask and diluted it with 50% methanol to scale, and then shook it well. The resulting solution was centrifuged at 13,000 r/min for 10 min, and an appropriate amount of supernatant was taken and passed through a 0.22 μm microporous filter membrane to obtain the test solution.

#### Instrument parameters by UPLC-Q-TOF–MS

Chromatographic separation was carried out on an Agilent Zorbax Eclipse Plus C_18_ RRHD (3.0 × 100 mm, 1.8 μm). The mobilephase was composed of 0.1% formic acid (FA) solution (A) and acetonitrile (B), with a gradient elution procedure as follows: 0–1 min, 5%B; 1–15 min, 50%B; 15–35 min, 95%B; 35–38 min, 95%B; 38–38.5 min, 5%B; 38.5–42 min, 5%B. The flow rate was 0.30 mL/min, and the column temperature was set at 40 °C, and the injection volume was 2 μL. The instrumental settings of UPLC-Q-TOF–MS were as follows: an electrospray ion source (ESI) was used in positive and negative ion modes, and the scanning range was m/z 50–1500. The positive and negative ion mode parameters were as follows: the spray gas pressure was 40 psi. Temperature: 325 °C in ESI^+^ mode, 350 °C in ESI^−^ mode. N_2_ flow rate: 8 L/min in ESI^+^ mode, 11 L/min in ESI^+^ mode. Based on the above conditions, the compound structure of JE was inferred by combining the precise molecular weight of MS, references, secondary map matching of the CDL database and MSC software.

### Network pharmacology analysis

#### Target prediction

According to the results of UPLC-Q-TOF–MS analysis and inflammation from the literature, ingredients were imported into the Swiss ADME website (http://www.swissadme.ch/index.php) and TCMSP database (https://tcmsp-e.com/tcmsp.php) in the form of SMILES numbers for active ingredient screening. Components with at least two “yes” among high GI absorption and Lipinski, Ghose, Veber, Egan and Muegge in drug-likeness from ADME or “OB% > 30 and DL > 0.18”^32–35^ from TCMSP were regarded as active compounds. JE active components were used for target prediction using the Swiss Target Prediction online tool (http://www.swisstargetprediction.ch/). “Homo sapiens” was selected for the species, and screening was performed according to “Probability > 0.1” to obtain the active ingredient targets of active compounds. The targets related to UC were collected by using the keyword “Ulcerative Colitis” in the databases of CTD (https://ctdbase.com/) and Gene Card (https://www.genecards.org/), and the targets related to UC were selected according to the “Inference Score > 10”. The Venny 2.1.0 tool (https://bioinfogp.cnb.csic.es/tools/venny/) was used to determine the common target Venn diagram of the intersection of JE treatment UC. The PPI network was built by the STRING online tool (https://string-db.org/).

#### KEGG pathway enrichment analysis

The Kyoto Encyclopedia of Genes and Genomes (KEGG, https://www.genome.jp/kegg/)^36–38^ is a database for systematic analysis functions associated with the target, which includes the latest the potential biological pathways. KEGG enrichment analysis can obtain the functional information of massive genes from a macro perspective, and predict underlying drug-disease signaling pathways. After obtaining the common targets, STRING processing were imported into the Metascape online tool (https://metascape.org/gp/index.html) for KEGG pathway analysis, and the relevant data of KEGG pathways were obtained. We selected the top twenty KEGG pathways with the smallest *p* value and import them into the bioinformatics mapping website (http://www.bioinformatics.com.cn/) to draw bubble graph.

Then, based on this basic pathway information, we are filtering out closely linked pathways related to UC pathology. This lays the foundation for subsequent integrating the results of network pharmacology and metabolomics and assembled an incorporated pathway for JE treatment of UC.

### Metabolomics analysis

#### Preparation of serum samples

Blood samples were left for 30 min at room temperature and centrifuged at 12,000 rpm for 15 min at 4 °C. Serum samples were collected and 100 μL serum was added to 400 μL of precooled acetonitrile and water mixture, placed in refrigerator for 1 h, and centrifuged for 15 min. The supernatant (400 μL) was blown dry with nitrogen. Then, 100 μL of precooled acetonitrile and water mixture was added, vortexed for the 30 s, and centrifuged for 15 min, and 80 uL of the supernatant was accurately absorbed into the injection bottle for sample analysis.

Quality control (QC) sample were obtained by mixing 10 μL of serum samples from the above mice, and the samples were prepared as described above. Before sample analysis, six QC samples were continuously tested. In the process of sample analysis, an AQC sample was added for every six samples to check the stability and repeatability of the system.

#### UPLC-Q-TOF–MS conditions

The chromatographic conditions were as follows: Waters UPLC BEH C18 column (2.1 mm × 100 mm, 1.7 μm), flow rate 0.3 mL·min^−1^, injector temperature 4 °C, column temperature 35 °C, injection volume 1 μL. Mobile phase: gradient elution of acetonitrile (A) − 0.1% formic acid aqueous solution (B) (0–2 min, 1% A; 2–8 min, 1–55% A; 8–17 min, 55–98% A; 17–20 min, 98% A; 20–20.5 min, 98–1% A; 20.5–25 min, 1% A).

MS conditions electrospray ion source (ESI), positive and negative ion scanning mode, scanning range m/z 50–1000, atomization gas pressure was set at 55 psi, curtain gas pressure was set at 35 psi, auxiliary gas pressure was set at 55 psi, atomization temperature was 500 °C. In negative ion mode, the capillary voltage was − 4.5 kV, the splitting voltage was − 60 V, the collision energy was 35 eV, and the collision energy superimposed was 15 eV. In the positive ion mode, the capillary voltage was 5.5 kV, the splitting voltage was 60 V, the collision energy was 35 eV, and the collision energy superimposed was 15 eV.

#### Data processing of metabolomics results

Unsupervised principal component analysis (PCA), supervised partial least discriminant analysis (PLS-DA) and orthogonal partial least discriminant analysis (OPLS-DA) models were used for multivariate statistical analysis by SIMCA 14.1 software. Then, the online tool Metaboanalyst 5.0 was used for the statistical analysis of univariate Student’s t test (T test), and finally, the difference multiple (FC value) analysis was performed. The metabolites (VIP > 1, *p* < 0.05, FC value < 0.8 or FC value > 1.2) that were significantly different between the model group and the control group and between the JE group and the model group were screened for preliminary identification and analysis. The changes in these metabolites were further analyzed. The metabolites that had significant differences between the JE group and the model group and had a recovery trend compared with the control group were screened as the differential metabolites for the treatment of UC.

#### Pharmacological verification

Immunohistochemistry for IκB, NF-κB p65 and COX-2 was performed on 3 mm thick paraffin-embedded sections from the colons of mice. First, the sections were dewaxed with environmentally friendly dewaxing solution, anhydrous ethanol and water. Second, the sections were repaired by using antigen repair solution, incubated in 3% hydrogen peroxide solution for 25 min, placed in PBS (pH 7.4), washed three times, and the tissue was covered uniformly by adding 3% BSA dropwise in the histochemical circle, which was closed at room temperature for 30 min. Third, the slides were incubated dropwise with primary antibody at 4 °C overnight, and secondary antibody was added to the slides and incubated at room temperature for 50 min. Then, the slides were washed with PBS (pH 7.4) by shaking and then added dropwise with fresh DAB chromogenic solution; the nuclei were stained with hematoxylin, dehydrated with different gradients of alcohol, air-dried and sealed with blocking gel. Finally, we examined all sections using a microscope.

#### Statistical analysis

All data were statistically analyzed with the SPSS 22.0 software. The Kruskal Wallis test and one-way ANOVA followed by LSD post hoc test were used to determine the significance of the differences between the groups. A value of *p* < 0.05 was considered statistically significant. All the results are expressed as the mean ± SD.

## Results

### Effect of JE on DSS induced UC mice

**Figure 2:**
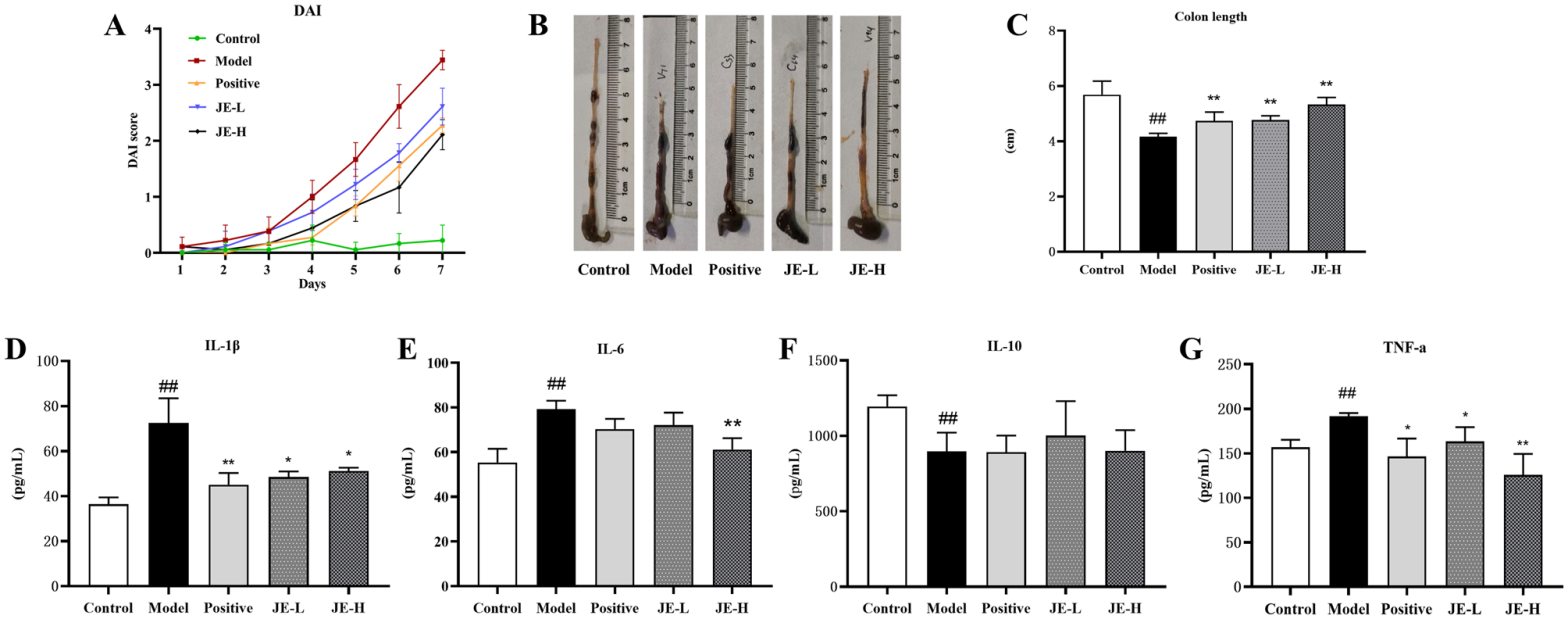
Effect of JE on UC mice induced by 2.5% DSS (*n* = 6). (**A**) Disease activity index (DAI) scores. (**B**) Representative colon from each. (**C**) Colon length. (**D**–**G**) The contents of IL-1β, IL-6, IL-10 and TNF-α in colon tissue by using ELISA kits. Data are shown as the mean ± SD. ^#^*p* < 0.05; ^##^*p* < 0.01, compared with the control group, *p < 0.05; **p < 0.01, compared with model group.

During the treatment period, the DAI was assessed as an indicator of the success of UC model construction (Fig. 2A). As expected, 2.5% DSS caused a rise in the DAI score (starting on Day 3). In addition, these changes were decreased by using the positive medicine mesalazine. The JE-H and JE-L groups were also significantly inhibited by DAI. Meanwhile, the colon length was observably shorter in the model group than in the control group, but this effect was markedly suppressed in the JE-L, JE-H and Positive groups (Fig. 2B,C). To analyze the effect of JE on the UC model of DSS-stimulated mice at a micro level, we measured the levels of IL-1β, IL-6, IL-10 and TNF-α in colon tissue using mouse-specific ELISA kits. As shown in Fig. 2D–G, compared with the control group, the anti-inflammatory factor IL-10 level was significantly decreased, and IL-1β, IL-6 and TNF-α pro-inflammatory cytokine levels were found to be markedly enhanced in the model group. The JE-L, JE-H, and positive groups significantly reversed these trends, except that they had no significant effect on IL-10 (Fig. 2E). Surprisingly, IL-10 did not show any effect against UC, suggesting that JE was not responsible for the effects of IL-10.

**Figure 3:**
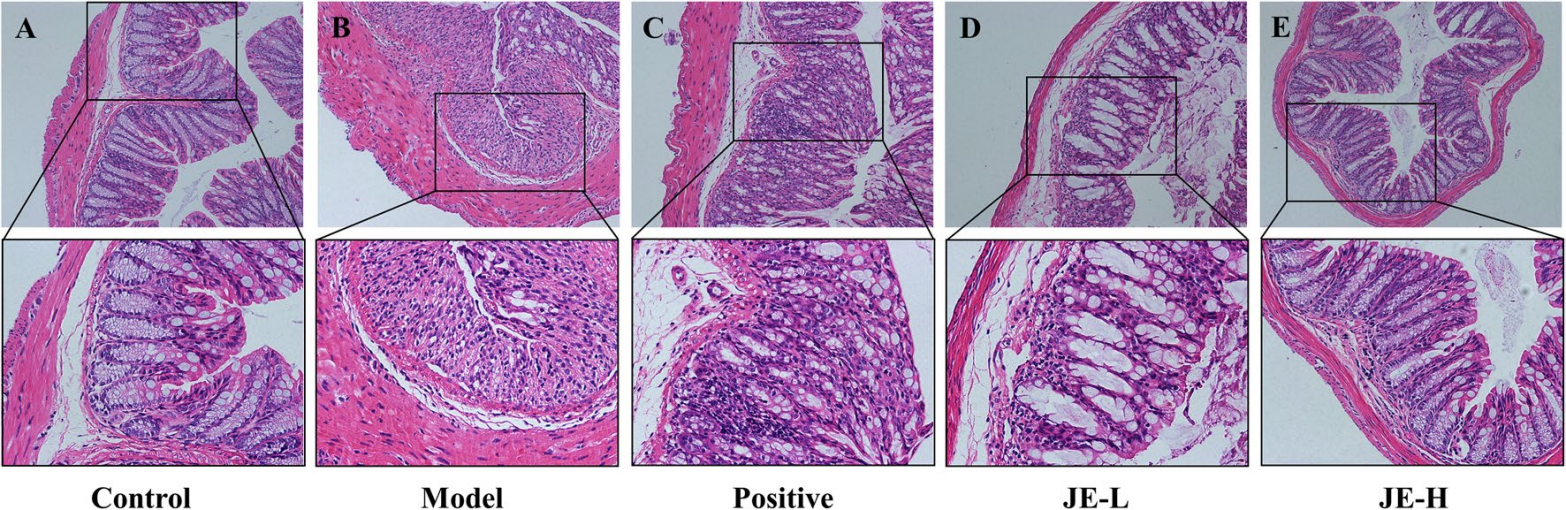
Representative H&E staining of colon tissues from the control group (**A**), model (2.5% DSS) group (**B**), positive group (**C**), JE-L group (**D**) and JE-H group (**E**) (*n* = 6).

Microscopic observation revealed that the colon tissue structure of mice in the control group had no obvious pathological morphology, with clear and complete distribution among the mucosal layer, submucosa layer, muscularis layer and serosa layer, and U-shaped crypts arranged neatly without obvious inflammatory cell infiltration. In contrast, mice in the model group exhibited obvious inflammatory cell (neutrophils and lymphocytes) infiltration, mucosal lamina propria erosion, severe or even absent crypt structure damage, goblet cell loss, and monolayer columnar epithelial cell injury and deformation in the colonic mucosa, submucosa, and muscularis. These effects were improved to varying degrees by administration of JE-L, JE-H, and mesalazine (Fig. 3). Overall, these results demonstrated the therapeutic effect of JE, especially JE-H, in UC mice.

### Material basis identification and active ingredients of JE

The chemical components of JE were identified and analyzed by UPLC-Q-TOF–MS method, and the total ion flow spectrum of JE is shown in Supplementary Fig. 1. Fifty-six compounds were identified based on the mass spectrometry data analysis of compound retention time, accurate relative molecular mass and other information (Table 2). The chemical compounds of JE were mainly terpenoids and organic acids. Based on the conditions specified above, combined with literature reports, a total of 25 active ingredients with high gastrointestinal absorption and drug-like properties were screened out (Table 3).

**Table 2:** Identification of JE chemical components.

No.	Compound	t_R_/min	Experimental mass (m/z)	Formula	Error	Category
1	Mannitol	1.577	181.0714[M-H]^−^	C_6_H_14_O_6_	− 1.72	Others
2	Anhydrous glucose	1.585	179.0562[M-H]^−^	C_6_H_12_O_6_	0.27	Amino Acids
3	Stachydrine	1.676	144.1019[M+H]^+^	C_7_H_13_NO_2_	0.24	Alkaloids
4	L-leucine	1.718	132.1021[M+H]^+^	C_6_H_13_ NO_2_	1.78	Amino Acids
5	Catalpol	3.598	407.1194[M+HCOO]^−^	C_15_H_22_O_10_	− 0.30	Terpenoids
6	Jasminanhydride	4.111	181.0506[M-H]^−^	C_9_H_10_O_4_	0.08	Terpenoids
7	Protocatechuic acid*	5.345	153.0194[M-H]^−^	C_7_H6O_4_	0.27	Organic acids
8	8-O-acetyl shanzhiside methyl ester	6.116	447.1501[M-H]^−^	C_19_H_28_O_12_	− 0.38	Terpenoids
9	8-O-Acetylharpagide	6.132	405.1403[M-H]^−^	C_17_H_26_O_11_	− 0.81	Terpenoids
10	Epikingisidic acid	6.339	389.1088[M-H]^−^	C_16_H_22_O_11_	− 0.26	Terpenoids
11	Chlorogenic acid*	6.646	353.0875[M-H]^−^	C_16_H_18_O_9_	− 0.63	Organic acids
			355.1026[M+H]^+^	C_16_H_18_O_9_	0.74	
12	Salicylic acid*	6.654	137.0242[M-H]^−^	C_7_H_6_O_3_	− 1.52	Organic acids
13	Dehydrologanin	7.06	433.135[M+HCOO]^−^	C_17_H_24_O_10_	− 0.20	Terpenoids
			389.145[M+H]^+^	C_17_H_24_O_10_	0.65	
14	Loganin	7.267	389.1449[M-H]^−^	C_17_H_26_O_10_	− 1.25	Terpenoids
15	Octahydro-6-hydroxy-7-methyl-1-oxocyclopenta[c]pyran-4-carboxylic acid	7.267	229.1069[M+H^+^	C_11_H_16_O5	− 0.65	Others
16	Rhapontin	7.325	465.1399[M+HCOO]^−^	C_21_H_24_O_9_	− 0.97	Others
17	Curculigoside	7.325	465.1398[M-H]^−^	C_22_H_26_O_11_	− 0.95	Terpenoids
18	Caffeic acid*	7.391	179.0347[M-H]^−^	C_9_H_8_O_4_	− 0.92	Organic acids
19	Deacetyl asperulosidic acid methyl ester	7.524	403.1245[M-H]^−^	C_17_H_24_O_11_	− 0.21	Terpenoids
20	Cantharidin	7.524	197.0809[M+H]^+^	C_10_H_12_O_4_	0.21	Terpenoids
21	Bilobalide	8.211	371.0985[M+HCOO]^−^	C_15_H_18_O_8_	0.28	Terpenoids
22	Ethyl caffeate*	8.377	209.0810[M+H]^+^	C_11_H_12_O_4_	0.46	Terpenoids
23	Schaftoside	8.634	609.1455[M+HCOO]^−^	C_26_H_28_O_14_	− 1.09	Flavonoid
24	Rutin*	8.634	609.1455[M-H]^−^	C_27_H_30_O_16_	− 0.99	Flavonoid
			611.1611[M+H]^+^	C_27_H_30_O_16_	0.53	
25	Benzoic acid	9.214	121.0298[M-H]^−^	C_7_H_6_O_2_	− 0.38	Organic acids
26	Griffonilide	9.214	167.035[M-H]^−^	C_8_H_8_O_4_	− 0.25	Lactones
27	Glucosylvitexin	9.33	593.1508[M-H]^−^	C_27_H_30_O_15_	− 0.81	Flavonoid
28	1,3-O-Dicaffeoylquinic acid	9.454	515.1188[M-H]^−^	C_25_H_24_O_12_	− 1.26	Others
			517.134[M+H]^+^	C_25_H_24_O_12_	− 0.11	
29	Scopoletin*	9.595	193.0496[M+H]^+^	C_10_ H_8_ O_4_	0.62	Lactones
30	Forsythin	9.785	579.2072[M+HCOO]^−^	C_27_H_34_O_11_	− 2.18	Lignanoids
31	Momordin IC	11.243	809.4316[M+HCOO]^−^	C_41_H_64_O_13_	− 1.64	Terpenoids
32	Cinnamic acid	11.392	193.0504[M+HCOO]^−^	C_9_H_8_O_2_	− 1.27	Organic acids
			149.0597[M+H]^+^	C_9_H_8_O_2_	− 0.25	
33	Isoferulic acid	11.392	193.0504[M-H]^−^	C_10_ H_10_ O_4_	− 0.97	Organic acids
34	Asiaticoside	12.096	957.5039[M-H]^−^	C_48_H_78_O_19_	− 1.81	Terpenoids
35	Raddeanin A	12.576	941.5107[M+HCOO]^−^	C_47_H_76_O_16_	− 1.28	Terpenoids
36	Liquidambaric acid	12.924	455.3523[M+H]^+^	C_30_H_46_ O_3_	0.50	Terpenoids
37	Esculentoside A	12.982	825.4268[M-H]^−^	C_42_H_66_O_16_	− 1.29	Terpenoids
38	Chikusetsusaponin IVa	13.032	793.4368[M-H]^−^	C_42_H_66_O_14_	− 1.42	Terpenoids
39	Alantolactone	15.094	277.144[M+HCOO]^−^	C_15_H_20_O_2_	− 1.28	Terpenoids
40	Saikosaponin A	16.42	825.4629[M-H]^−^	C_42_H_68_O_13_	− 1.87	Terpenoids
41	12-Hydroxyjasmonic acid lactone	16.544	207.1026[M-H]^−^	C_12_ H_16_O_3_	− 0.10	Others
42	Ethyl-p-methoxycinnamate	16.577	251.0925[M+HCOO]^−^	C_12_H14O_3_	0.58	Others
43	Dehydrocostuslactone	16.71	231.1382[M+H]^+^	C_15_H_18_O_2_	0.94	Terpenoids
44	Anise oil/cis-Anethole	16.767	193.0871[M+HCOO]^−^	C_10_H_12_O	0.25	Others
45	Ecliptasaponin A	17.215	633.3963[M-H]-	C_36_ H_58_O_9_	− 1.23	Terpenoids
46	Aloeemodin	20.876	269.0452[M-H]^−^	C_15_H_10_O_5_	− 0.91	Anthraquinones
47	Germacrone	21.795	219.1745[M+H]^+^	C_15_H_22_O	0.59	Terpenoids
48	Magnolol	23.228	265.1231[M-H]^−^	C_18_ H_18_ O_2_	− 1.41	Lignanoids
49	Recibufogenin	23.51	383.2224[M-H]^−^	C_24_H_32_O_4_	− 1.03	Others
50	Hederagenin	24.363	471.3475[M-H]^−^	C_30_H_48_O_4_	− 1.08	Terpenoids
51	α-Linolenic acid	24.553	279.231[M+H]^+^	C_18_H_30_O_2_	0.67	Organic acids
52	Curdione	26.293	237.1852[M+H]^+^	C_15_H_24_O_2_	1.22	Terpenoids
53	Betulinic acid	31.345	455.3529[M-H]^−^	C_30_H_48_O_3_	− 0.51	Terpenoids
54	Alisol B 23-acetate	32.969	513.3584[M-H]^−^	C_32_H_50_O_5_	− 0.33	Terpenoids
55	Malvalic acid	33.441	279.2332[M-H]^−^	C_18_H_32_O_2_	0.72	Others
56	Palmitic acid	35.296	255.2331[M-H]^−^	C_16_H_32_O_2_	0.52	Organic acids

*Identified by comparison with reference standards.

**Table 3:** Active chemical components of JE.

No.	Compound	CAS no.	Formula
1	L-leucine	61-90-5	C_6_H_13_ NO_2_
2	Ethyl caffeate	102-37-4	C_11_H_12_O_4_
3	Magnolol	528-43-8	C_18_ H_18_ O_2_
4	Dehydrocostus lactone	477-43-0	C_15_H_18_O_2_
5	Caffeic acid	331-39-5	C_9_H_8_O_4_
6	Alantolactone	546-43-0	C_15_H_20_O_2_
7	Cantharidin	56-25-7	C_10_H_12_O_4_
8	Isoferulic acid	537-73-5	C_10_ H_10_ O_4_
9	Griffonilide	61371-55-9	C_8_H_8_O_4_
10	Curdione	13657-68-6	C_15_H_24_O_2_
11	Hederagenin	465-99-6	C_30_H_48_O_3_
12	Alisol B 23-acetate	26575-95-1	C_32_H_50_O_5_
13	Salicylic acid	69-72-7	C_7_H_6_O_3_
14	1,3-O-Dicaffeoylquinic acid	30964-13-7	C_25_H_24_O_12_
15	Benzoic acid	65-85-0	C_7_H_6_O_2_
16	Malvalic acid	503-05-9	C_18_H_32_O_2_
17	Scopoletin	92-61-5	C_10_ H_8_ O_4_
18	Protocatechuic acid	99-50-3	C_9_H_10_O_4_
19	Recibufogenin	465-39-4	C_24_H_32_O_4_
20	Ferulic acid	1135-24-6	C_10_ H_10_ O_4_
21	Methyl caffeate	3843-74-1	C_10_ H_10_ O_4_
22	Isochlorogenic acid B	14534-61-3	C_25_H_24_O_12_
23	4-hydroxyphenylacetic acid	156-38-7	C_8_H_8_O_3_
24	(+)-Lariciresinol	27003-73-2	C_20_H_24_O_6_
25	Patriscabratine	-	C_27_H_28_O_4_N_2_

### Network-based analysis of JE’s effects and mechanism

#### Putative targets of JE and UC

**Figure 4:**
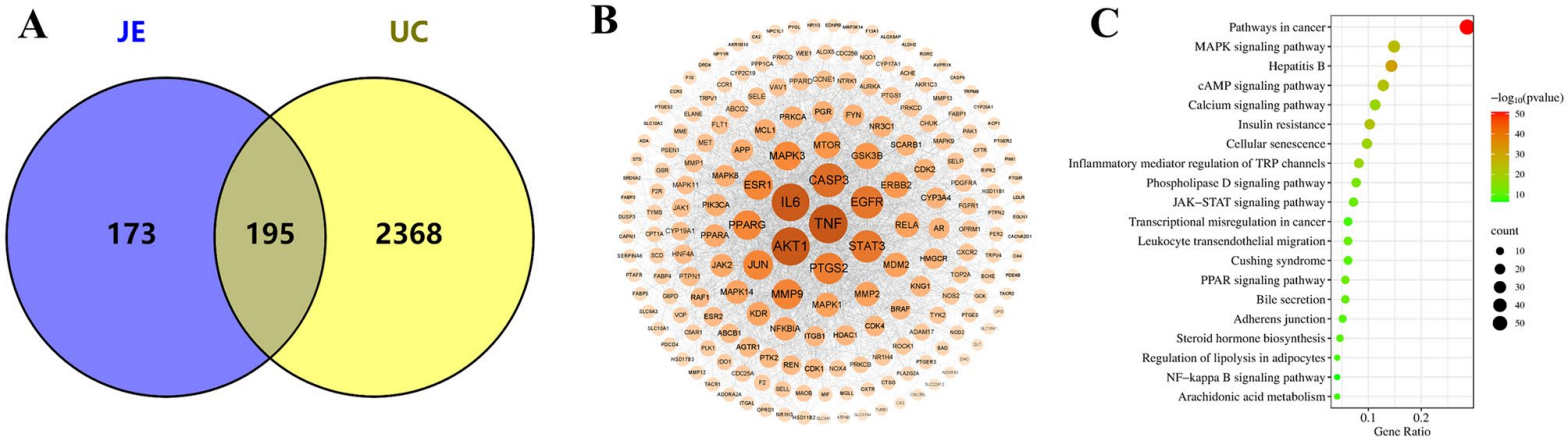
(**A**) Venn diagram for JE and UC. (**B**) Common targets used to establish the PPI network. C. KEGG enrichment analysis of common targets for UC treated with JE.

To identify the putative targets of JE's 25 active ingredients, we used the Swiss Target Prediction platform and obtained JE targets using “Probability > 0.1” as the screening criterion. After removing duplicates, 368 targets were obtained. We then used the CTD and Gene Cards databases to screen for UC-related action targets with “Inference Score > 10” as the filter threshold and removed duplicates, resulting in 2563 targets. After matching UC targets and active ingredient targets, a total of 195 potential targets related to JE in the treatment of UC were identified in Venny 2.1 (Fig. 4A).

#### PPI network and KEGG pathway analysis

To understand the complex interaction of common targets at a systems level, the 195 potential targets were imported into the STRING online tool for PPI network analysis, which contained 195 nodes, 2564 edges, 26.3 average nodal points and average local clustering coefficient of 0.524. The common targets were visualized in a PPI network diagram based on the degree value size by Cytoscape 3.9.1 (Fig. 4B). As the degree of the target increases, the nodes become larger, and the color changes from yellow to orange-red. Twenty-five shared targets were chosen as potential core targets, including TNF, AKT1, IL6, CASP3, EGFR, STAT3, PTGS2, MMP9, JUN, ESR1, PPARG, MAPK3, GSK3B, MTOR, ERBB2, MDM2, RELA, MMP2, MAPK1, KDR, MAPK14, NFKBIA, JAK2, PPARA and PIK3CA.

We further analyzed the 195 potential targets using the Metascape online tool for KEGG enrichment analysis, and 172 signaling pathways were enriched. We ranked the top 20 signaling pathways using bubble plots for visual analysis (Fig. 4C), and after analysis, we determined that the most relevant pathways to UC were the NF-κB signaling pathway, arachidonic acid metabolism, JAK-STAT signaling pathway, and MAPK signaling pathway (Table 4).

**Table 4:** KEGG pathway of JE on UC.

ID	Pathway	P value	Gene	Gene
hsa04064	NF-kappa B signaling pathway	3.87E-7	8	TNF, PTGS2, RELA, NFKBIA, CHUK, PRKCB, PRKCQ, MAP3K14
hsa00590	Arachidonic acid metabolism	5.60E-9	8	PTGS2, PTGS1, ALOX5, CYP2C19, AKR1C3, PTGES, PTGES2, PLA2G2A,
hsa04630	JAK-STAT signaling pathway	4.37E-12	14	IL6, AKT1, STAT3, EGFR, MTOR, JAK1, JAK2, MCL1, PDGFRA, PIK3CA, PIM1, PTPN2, RAF1, TYK2
hsa04010	MAPK signaling pathway	1.58E-25	29	TNF, RELA, MAPK14, AKT1, BRAF, CACNA2D1, CASP3, CDC25B, CHUK, DUSP3, EGFR, ERBB2, FGFR1, FLT1, JUN, KDR, MET, NTRK1, PAK1, PDGFRA, PRKCA, PRKCB, MAPK1, MAPK3, MAPK8, MAPK11, MAPK9, RAF1, MAP3K14

### Detection of differential metabolites in mouse serum

To determine the differential metabolites of UC mice by JE treatment, we chose the JE-H group with the best efficacy for metabolomic analysis based on the pharmacodynamic experiment. Serum metabolites were measured in the control, model and JE-H groups using an untargeted metabolomics approach (Tables 5, 6, 7, 8).

**Figure 5:**
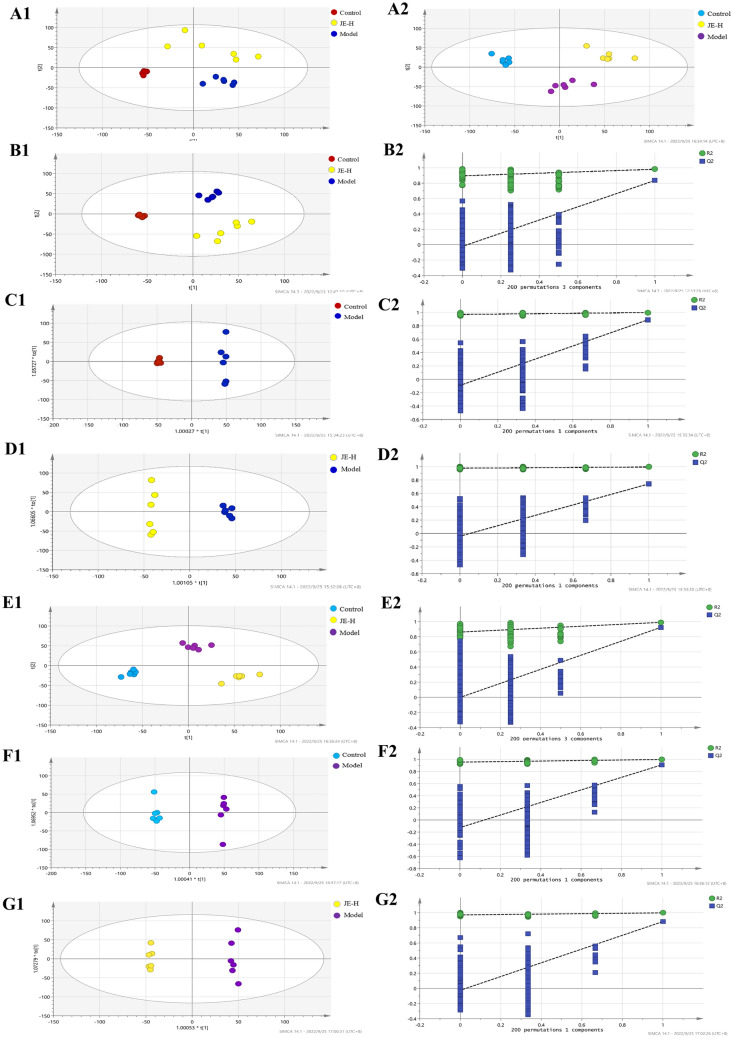
PCA score plot (**A1**: ESI^−^, **A2**: ESI^+^); PLS-DA score plot (**B1**, **E1**); 200 permutation tests of PLS-DA score plot (**B2**, **E2**); OPLS-DA score plots of Model group and Control group (**C1**, **F1**); 200 permutation tests of OPLS-DA score plot of Model group and Control group (**C2**, **F2**); OPLS-DA score plots of JE-H group and Model group (**D1**, **G1**); 200 permutation tests of OPLS-DA score plot of JE-H group and Model group (**D2**, **G2**) in negative (**B**,**C**,**D**) and positive (**E**,**F**,**G**) ESI mode (*n* = 6).

The obtained data were analyzed using multivariate statistical methods, including PCA, PLS-DA, and OPLS-DA, under positive and negative ion modes. QC samples demonstrated good stability of the system. The total ion chromatograms of the control, model and JE-H groups were shown in the supplemental materials. The PCA score plots (Fig. 5A1,A2) showed that the three groups were clearly separated from each other, for both the ESI^+^ and ESI^−^ modes. This indicated a significant change in metabolites in the different groups, consistent with the appearance of physiological indicators. The supervised statistical PLS-DA model plots for the three groups in ESI^+^ and ESI^−^ modes were obviously distinguishable. Under ESI^−^ mode the parameters of R^2^ and Q^2^ in PLS-DA were 0.987 and 0.862 in serum samples (Fig. 5B1). Under ESI^+^ mode, the parameters of R^2^ and Q^2^ in PLS-DA were 0.987 and 0.885 in serum samples (Fig. 5E1). As shown in Fig. 5B2,D2, the models had good R^2^ and Q^2^ values of 200 permutation tests, indicating that the PLS-DA models were reliable with a low risk of overfitting in ESI^+^ and ESI^−^ modes (Tables 6, 8).

In addition, we found that in ESI^+^ and ESI^−^ mode, the OPLS-DA models were significantly different in the samples between the control group compared to the model group and the model group compared to the JE-H group, and all samples were located within the 95% confidence interval (Tables 5, 7 and Fig. 5C1–G1). It indicated that the levels of certain metabolites in the mice changed under pathological conditions. In the OPLS-DA models, R^2^and Q^2^ were satisfied (Tables 6, 8 and Fig. 5C2–G2). The results of the permutation test indicate that the model was stable and dependable and could be used for further data analysis.

**Table 5:** The parameters of PLS-DA and OPLS-DA models.

ESI^−^	PLS-DA	OPLS-DA	
	SUM	Control and model	Model and JE-H
R^2^	0.987	0.998	0.995
Q^2^	0.862	0.890	0.741

**Table 6:** The parameters of the 200 permutations test.

ESI^−^	PLS-DA	OPLS-DA	
	SUM	Control and model	Model and JE-H
R^2^	0.894	0.969	0.976
Q^2^	− 0.022	− 0.090	− 0.040

**Table 7:** The parameters of PLS-DA and OPLS-DA models.

ESI^+^	PLS-DA	OPLS-DA	
	SUM	Control and model	Model and JE-H
R^2^	0.987	0.997	0.908
Q^2^	0.885	0.997	0.882

**Table 8:** The parameters of the 200 permutations test.

ESI^+^	PLS-DA	OPLS-DA	
	SUM	Control and Model	Model and JE-H
R^2^	0.859	0.953	0.970
Q^2^	− 0.039	− 0.126	− 0.025

R^2^ represents the goodness of fit of models; Q^2^ represents the predictability of models; SUM represents compare control group, model group with JE-H group; Control and Model represents compare control group with model group; Model & JE-H represents compare model group with JE-H group.

**Figure 6:**
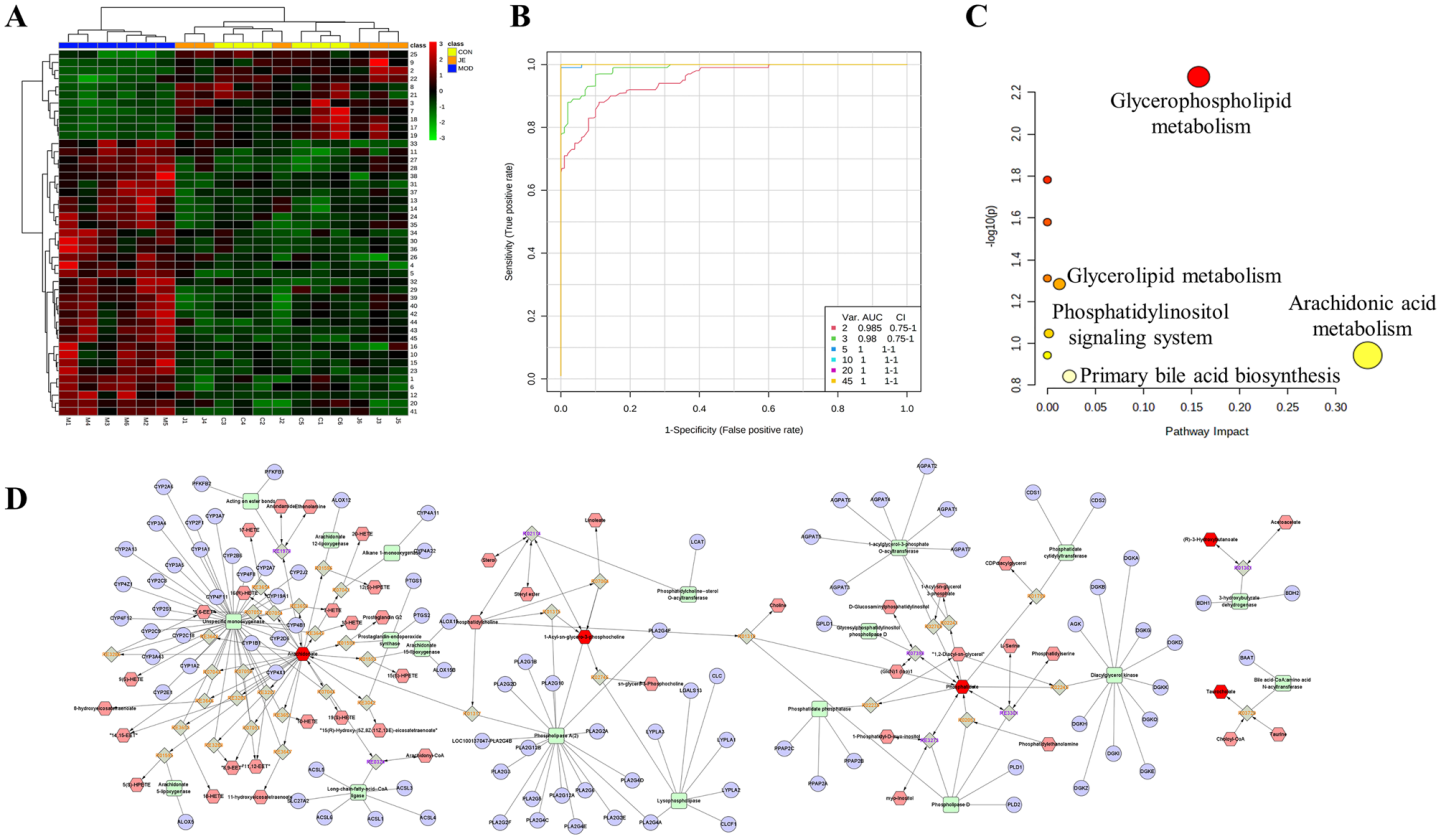
(**A**) Heatmap analysis of 45 biomarkers. (**B**) ROC curves of the control and model groups. (**C**) Pathway analysis of biomarkers disturbed by UC treated with JE. (**D**) “Compound-Reaction-Enzyme-Gene” network (Red hexagon: metabolite; Orange prismatic: enzymatic reaction; Green square: reaction-related enzyme; Blue circle: Gene).

With a threshold of “VIP > 1, *p* < 0.05, FC < 0.8 or FC > 1.2”, the metabolites were identified between the model group and the control group, and between the JE group and the model group. The results of this experiment yielded 45 potential biomarkers that could be potential for JE for UC (Table 9). To demonstrate the levels of variation of the 45 differential metabolites between the model group and the control group and between the administration group and the model group, this experiment was visualized using a clustering heatmap (Fig. 6A). In the clustering heatmap, the model group clustered into one category, and the control group and the drug administration group clustered into another category, indicating that the model group differed more than the control group. The clustering of the administered group with the control group into one category indicates that the number of expression trends of these differential metabolites are relatively consistent and well correlated.

**Table 9:** Differential metabolites of each group.

No.	Identification	MW	RT (min)	Iron Mode	Model/control	JE/Model
1	P-xylene	107.0852	14.4087	[M+H]^+^	↑	↓
2	Cinnamic acid	131.0487	6.9346	[M+H]^+^	↓	↑
3	5-Hydroxyindole	134.0594	5.7819	[M+H]^+^	↓	↑
4	(+)-Pulegone	135.1165	15.6783	[M+H]^+^	↑	↓
5	Butylbenzene	135.1164	16.0706	[M+H]^+^	↑	↓
6	4-Propylphenol	137.0957	7.2539	[M+H]^+^	↑	↓
7	MLS000575022-01	225.1098	7.4197	[M+H]^+^	↓	↑
8	Pirlindole mesylate	227.1640	9.0905	[M+H]^+^	↓	↑
9	O-1918	287.1955	1.0157	[M+H]^+^	↓	↑
10	Aspartame	295.1290	5.5636	[M+H]^+^	↑	↓
11	Ajmaline	327.2171	15.8019	[M+H]^+^	↑	↓
12	Rimexolone	371.2583	8.6013	[M+H]^+^	↑	↓
13	5-hydroxy-2,2-dimethyl-7,10-bis(2-methylbut-3-en-2-yl)pyrano[3,2-g]chromen-8-one	381.2053	16.3180	[M+H]+	↑	↓
14	HTMT	383.2012	16.3198	[M+H]^+^	↑	↓
15	N-stearoyl tyrosine	448.3429	11.7405	[M+H]^+^	↑	↓
16	Cer-AS d27:4	450.3497	11.7665	[M+H]^+^	↑	↓
17	Strophanthidin Semicarbazide	462.2681	7.8092	[M+H]^+^	↓	↑
18	Cytochalasin B	480.2789	7.7958	[M+H]^+^	↓	↑
19	Taurocholic acid	516.3005	7.8138	[M+H]^+^	↓	↑
		514.2841	7.8508	[M-H]^−^	↓	↑
20	Scutellarein 6,4'-dimethyl ether 7-(6''-acetylglucoside)	519.1399	21.7285	[M+H]^+^	↑	↓
21	Calcimycin	524.2779	9.5045	[M+H]^+^	↓	↑
22	Fumitremorgin A	580.2942	12.0851	[M+H]^+^	↓	↑
23	Lanceotoxin A	621.2857	11.3963	[M+H]^+^	↑	↓
24	3-Hydroxybutyrate	103.0399	2.9730	[M-H]^−^	↑	↓
25	Ethyl everninate	209.0795	6.9414	[M-H]^−^	↓	↑
26	Ellipticine	245.1143	4.8547	[M-H]^−^	↑	↓
27	Arachidonic acid	303.2329	15.8049	[M-H]^−^	↑	↓
28	Icosatrienoic acid	305.2400	15.8063	[M-H]^−^	↑	↓
29	Glutamyltyrosine	309.1094	4.9120	[M-H]^−^	↑	↓
30	3α,7α,12β-Trihydroxy-5β-cholanate	407.2802	8.3662	[M-H]^−^	↑	↓
31	LPA (18:2(9Z,12Z)/0:0)	433.2375	21.7105	[M-H]^−^	↑	↓
32	FAHFA 29:6	455.3181	10.9142	[M-H]^−^	↑	↓
33	2-(Methoxycarbonyl)-5-methyl-2,4-bis(3-methyl-2-butenyl)-6-(2-methyl-1-oxopropyl)-5-(4-methyl-3-pentenyl) cyclohexanone	457.3343	12.3855	[M-H]^−^	↑	↓
34	(17α,23S)-Epoxy-28,29-dihydroxy-27-norlanost-8-ene-3,24-dione	471.3120	8.6663	[M-H]^−^	↑	↓
35	1-Hydroxy-2-(9Z,12Z,15Z-octadecatrienoyl)-sn-glycero-3-phosphoethanolamine	474.2638	10.7485	[M-H]^−^	↑	↓
36	Lucidenic acid G	475.2684	8.3518	[M-H]^−^	↑	↓
37	LPA (22:6)	481.2372	13.5786	[M-H]^−^	↑	↓
38	LPG 18:1	509.2905	15.1871	[M-H]^−^	↑	↓
39	LPC (20:4(8Z,11Z,14Z,17Z))	588.3314	11.6640	[M-H]^−^	↑	↓
40	LPC (20:3(5Z,8Z,11Z))	590.3467	12.2839	[M-H]^−^	↑	↓
41	LPI 20:4	619.2895	11.6448	[M-H]^−^	↑	↓
42	LPI 20:3	621.3058	13.0770	[M-H]^−^	↑	↓
43	LPI 22:6	643.2906	11.9968	[M-H]^−^	↑	↓
44	LPI 22:5	645.3083	13.4158	[M-H]^−^	↑	↓
45	LPI 22:4	647.3227	14.4554	[M-H]^−^	↑	↓

The results of the ROC curve exploration analysis showed "AUC > 0.9", which combined with the sensitivity and specificity showed a high accuracy of the biomarker (Fig. 6B). Metabolic pathway enrichment analysis of 45 differential metabolites was performed using the MetaboAnalyst 5.0 online tool, and the results were visualized as bubble plots (Fig. 6C). The results showed that the critical metabolic pathways of metabolites regulated by JE treatment of UC were arachidonic acid metabolism, glycerophospholipid metabolism and primary bile acid biosynthesis (Table 10). In order to explore the mechanism of JE in the treatment of UC, we imported differential metabolites into the Metascape plug-in of Cytoscape software and constructed a“Compound-Reaction-Enzyme-Gene” network (Fig. 6D). The network shows the process of biological information transfer from genes to metabolites. Among them, the arachidonic acid metabolic pathway showed that PTGS2 acted on arachidonic acid by reaction-associated enzymes and enzymatic reactions, which suggested that PTGS2 might be a key target of this metabolic pathway.

**Table 10:** Differential metabolite pathway enrichment analysis of UC on JE.

No.	Pathway name	p	− log(p)	Impact
1	Arachidonic acid metabolism	0.1141	0.9428	0.3329
2	Glycerophospholipid metabolism	0.0053	2.2747	0.1572
3	Primary bile acid biosynthesis	0.1439	0.8421	0.0229
4	Glycerolipid metabolism	0.0521	1.2834	0.0125
5	Phosphatidylinositol signaling system	0.0897	1.0473	0.0015
6	Synthesis and degradation of ketone bodies	0.0165	1.7822	0.0000
7	Taurine and hypotaurine metabolism	0.0263	1.5798	0.0000
8	Butanoate metabolism	0.0489	1.3108	0.0000
9	Biosynthesis of unsaturated fatty acids	0.1141	0.9428	0.0000

### Integrating the research results of metabolomics and network pharmacology

**Figure 7:**
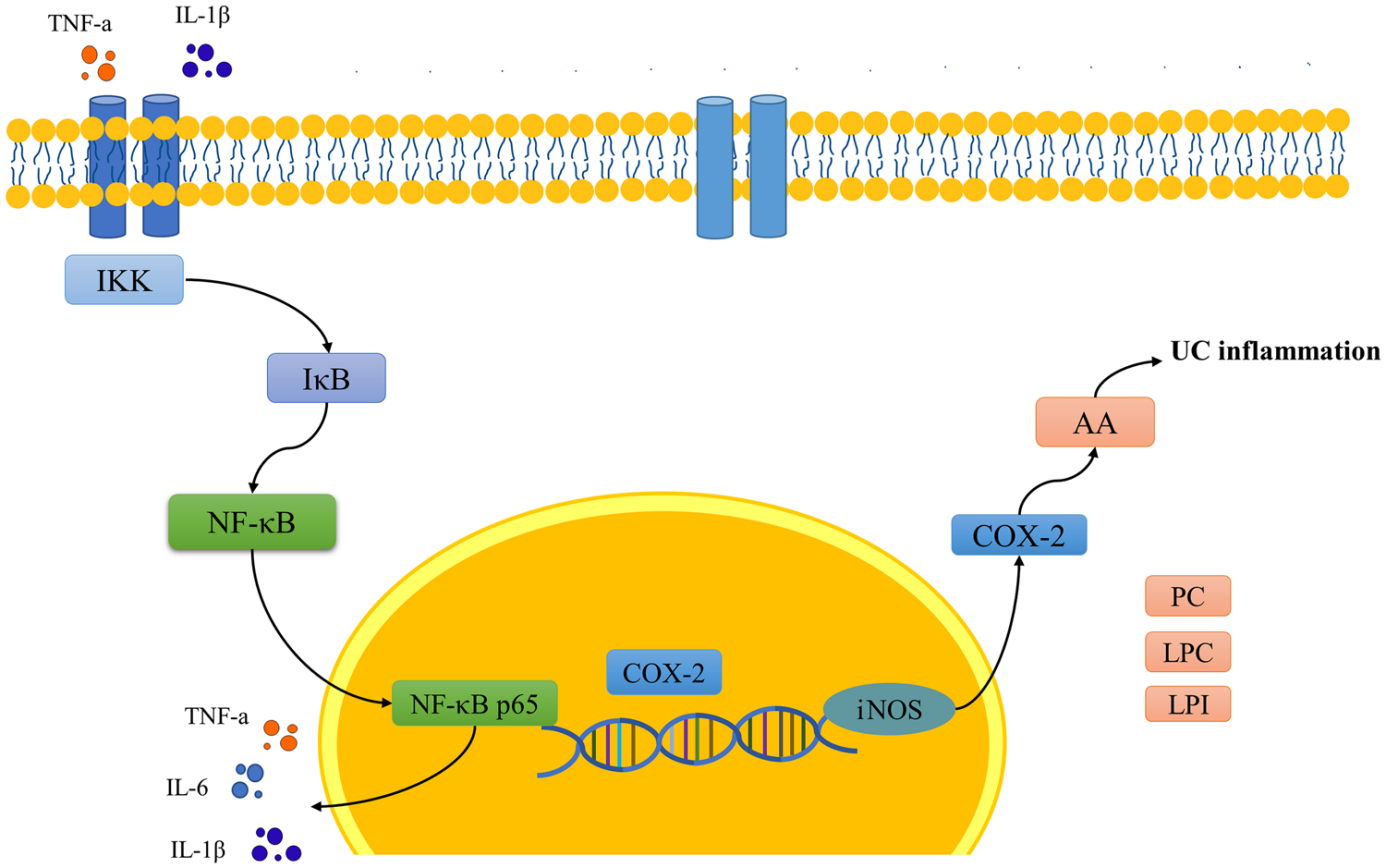
The mechanism map by integrated metabolomics and network pharmacology for JE treatment in UC.

In inflammatory response, NF-κB has been shown to possess two binding sites on COX-2 promoter region^39^. The transcription and synthesis of COX-2 are mainly regulated by NF-κB pathway^40^. The activated NF-κB pathway promotes the expression of various pro-inflammatory genes such as TNF-α, IL-6, IL-1β and the pro-inflammatory enzyme COX-2, influencing the course of mucosal inflammation. Furthermore, COX-2 was the key enzyme that catalyzed the biosynthesis of prostaglandins from arachidonic acid^41^. The research scholar verified the critical role of COX-2-mediated arachidonic acid metabolism in esophageal injury in rats by metabolomics techniques^42^. Importantly, we found that the arachidonic acid metabolic pathway was enriched by network pharmacology and metabolomics. PTGS2 (also known as COX-2), a key regulator of arachidonic acid metabolism, may be a crucial target for JE treatment of UC based on potential substance-based network pharmacological predictions and the results of metabolomics experiments. The COX-2 promoter contains the binding site of NF-κB, and p65 (RELA) is the core target screened by network pharmacology. To explore the integral regulation of JE for the treatment of UC, we assembled an integrated pathway by the upstream and downstream relationship (Fig. 7). In this pathway, JE can alleviate DSS-induced UC mainly by inhibiting the upstream pathway induced activation of NF-κB p65 and the downstream pathway induced the release of AA. Specifically, JE can decrease the levels of pro-inflammatory cytokines IL-1β, IL-6 and TNF-α. Blocking IκB was degraded after phosphorylation by IKK, which inhibited the release of NF-κB and is translocated to the nucleus, and then downregulated the expression of the iNOS gene through the transcription factor COX-2, downregulating the release of COX-2 and arachidonic acid (AA) to treat UC. Additionally, JE can inhibit the production of metabolites, which were including lysophosphatidylcholine (LPC), lysophosphatidylinositol (LPI) and other metabolites. And it is likely that JE exerts its anti-UC effects by inhibiting targets on this IκB/p65/COX-2 pathway. So that JE should not be ignored in treating UC.

**Figure 8:**
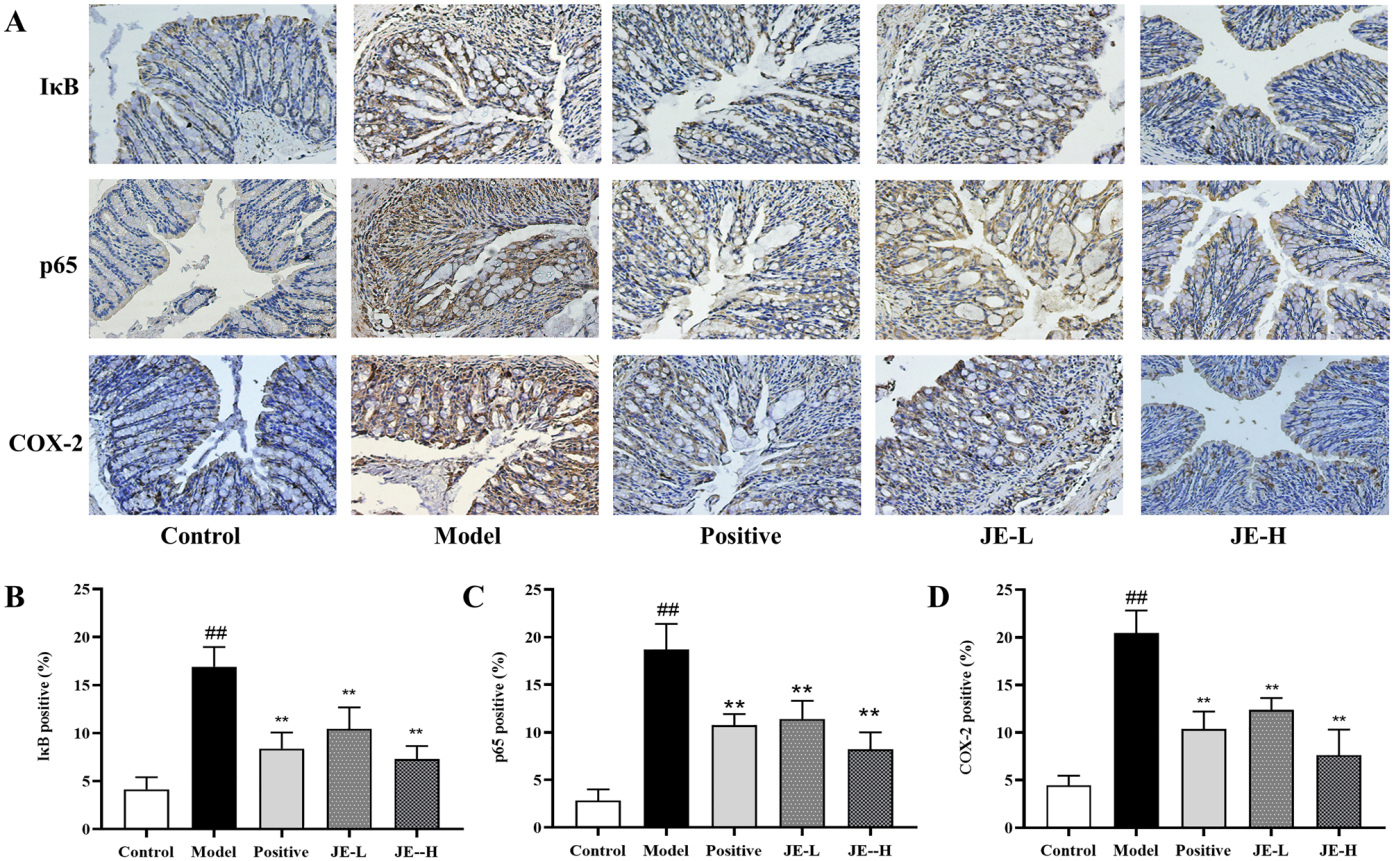
(**A**) Immunohistochemistry staining of colon tissues from the control group, model group, positive group, JE-L group and JE-H group (scale bar, × 200) (*n* = 6); (**B**–**D**). The contents of IκB, p65 and COX-2 positive in colon tissue by using IHC. Data are shown as the mean ± SD. ^#^*p* < 0.05; ^##^*p* < 0.01, compared with the control group, *p < 0.05; **p < 0.01, compared with model group.

The results of IHC showed that the expression of IκB, p65 and COX-2 was significantly increased in the colonic tissues of mice in the model group compared with the control group, while the expression of these proteins was markedly reduced in the drug administration group, especially in the JE-H group which was more significant (Fig. 8). These results suggest that UC can boost the activation of IκB/p65/COX-2 pathway, and JE reduces colonic inflammation and ameliorates UC by inhibiting the expression of these channel proteins.

## Discussion

JE is a characteristic common Chinese medicine from the Lingnan region of China, and the group's preliminary study showed that this medicine has significant anti-inflammatory effects and is often applied clinically to treat abdominal pain, diarrhea, and other gastrointestinal symptoms. Therefore, JE is likely to be a potential characteristic Chinese medicine for the treatment of UC. In this study, pharmacodynamics, network pharmacology and metabolomics were applied to conduct a preliminary exploration of the mechanism of action of JE for the treatment of UC. The ideas of network pharmacology and metabolomics are similar to the "holistic concept" of TCM. These two approaches are unique in predicting and investigating the mechanism of action of herbal medicines for the treatment of diseases with multi-component, multi-target, and multi-pathway characteristics.

### Biochemical analysis

UC is generally characterized by macroscopic features (e.g., elevated DAI scores and shortened colonic length), biochemical criteria (e.g., elevated levels of pro-inflammatory factors IL-1β, IL-6 and TNF-α and decreased levels of anti-inflammatory factor IL-10) and colonic histopathology (damage to the mucosal layer, submucosal U-shaped crypt and cup cells, etc.). In this study, we found that compared with the control group, the model group mice had an elevated DAI, significantly shorter colon length, decreased IL-10 levels, and increased IL-1β, IL-6 and TNF-α levels. Pathological changes in the histological structure of the colon were observed. Inflammatory cell infiltration, erosion of the mucosal lamina propria and muscle layer, severe damage or even loss of crypt structure, massive loss of cup cells, and damage and deformation of monolayer columnar epithelial cells were clearly observed in the colonic mucosa, submucosa and muscle layer. Compared with the Model group, the Positive, JE-L and JE-H groups were able to reverse this effect and improve the histopathological changes to varying degrees, except for IL-10, and the reversal effect was better in the JE-H group than in the JE-L group, indicating that JE-H, the anti-UC effect, was enhanced.

Other research^18–20^ has determined significant differences in DAI, colon length and analysis of colon pathological sections and expression levels of inflammatory factors (IL-1β, IL-6, IL-10 and TNF-α) in colonic tissues of UC mice compared with the control group, which is basically consistent with the results of this experiment, indicating that this experimental UC mouse model was successful and reliable. After the administration of JE intervention, all of these indicators showed different degrees of modulation, which indicates the efficacy of JE in the treatment of UC.

### Network pharmacology analysis

To overcome the limitation of a lack of JE chemical components retrieved from the network database, we identified 56 JE chemical components through UPLC-Q-TOF–MS in this study. These components provided data to investigate the potential mechanism of action of JE for UC treatment using a network pharmacological approach.

Network pharmacology was applied to screen the identified components for activity and predict their targets. Our results yielded 25 active ingredients, 195 targets, 25 key targets, and 4 key signaling pathways. Among the main active ingredients of JE, ethyl caffeate^43^, salicylic acid^44^, and scopoletin^45^ have inhibitory effects on inflammatory responses. PPI results demonstrated that 25 targets, such as TNF, AKT1, IL6, CASP3, EGFR, STAT3 and PTGS2 are core targets and play an important role in UC treatment. KEGG analysis revealed pathways related to UC remission mechanisms in JE: NF-κB signaling pathway^46–48^, arachidonic acid metabolism^49–51^, JAK-STAT signaling pathway^52^, and MAPK signaling pathway^53^. The results of the network pharmacology prediction were similar to those in the literature, indicating the scientific validity and reliability of the study method, which is conducive to the elucidation of the mechanism of action of JE in the treatment of UC.

### Metabolomics analysis

The serum composition can be greatly affected by drugs, making the assessment of serum metabolomics a useful tool for identifying changes in drug metabolism. Our results demonstrate that JE can effectively regulate 45 differential metabolites, including taurocholic acid, arachidonic acid, N-stearoyl tyrosine, LPC (20:4(8Z,11Z,14Z,17Z)), and LPI (20:4), which are mainly involved in arachidonic acid metabolism and glycerophospholipid metabolism. These findings are in agreement with previous research on the main metabolic pathways of herbal medicine for UC^54^. Arachidonic acid (AA) is an essential free fatty acid that plays a critical regulatory role in inflammation and immune response. Excessive activity of AA metabolites and cytokines in the intestine can cause impaired immune system function and increase the probability of intestinal carcinogenesis. Study has shown that excessive metabolism of AA metabolites can directly damage colonic tissues and exacerbate the inflammatory response, which is an important link in the pathogenesis of UC^55^. In contrast, our study found that AA level was significantly reduced after treatment with JE, which attenuated the colonic inflammatory response and damage, thus treating UC. Lysophospholipids are a group of bioactive, proinflammatory lipid molecules, that include lysophosphatidylcholine (LPC), lysophosphatidylinositol (LPI), and others. It was found^56^ that LPI and LPC regulate downstream genes of NF-κB p65 (RELA), such as IL-1β. We hypothesize that JE inhibits IL-1β, a downstream gene of NF-κB p65, through glycerophospholipid metabolism, by downregulating the levels of LPC and LPI, thus exerting treatment of UC.

### The internal mechanism of JE in the treatment of UC

The potential relationship between biomarkers and core genes was analyzed by integrating the results of network pharmacology and metabolomics. PTGS2 (COX-2) and PTGS1 were hypothesized to be related to JE based on their appearance in both metabolomics and network pharmacology. COX-2 is an inducible enzyme that mediates prostaglandin synthesis and plays a key role in the inflammatory response^57^. it is induced by proinflammatory cytokines at the site of inflammation and shows an upregulation trend in both inflammation and cancer^58^. Jiang^59^ used Western blotting to determine COX-2 expression in mouse colonic tissues and found significantly higher expression in UC mice. The metabolomic approach was used to screen for differential metabolites, and AA was found to be significantly elevated in the model group, which was consistent with the present experiment. Meanwhile, NF-κB, a nuclear receptor, has been reported to be closely associated with UC^60,61^.

NF-κB is involved in the process of immune regulation and inflammatory response. Unactivated NF-κB is present in the cytoplasm and binds to its inhibitor IκB. In the conventional activation pathway, IκB is degraded by IKK phosphorylation, and the released NF-κB is translocated to the nucleus and regulates COX-2 gene expression through transcription factors. Although the expression of COX-2, NF-κB and IκB has been previously reported in many inflammatory conditions, there is a paucity of data investigating their expression in JE treatment of UC.

Therefore, we used IHC technology to validate the core targets IκB, p65, and COX-2, further confirming the accuracy of biomarkers based on metabolomics technology. Combined with the data analysis of this experiment, JE can decrease the levels of IκB, which inhibited the release of p65 and downregulated the expression of COX-2, downregulating AA to treat UC. This study demonstrated that JE reverses 2.5% DSS-induced UC in mice via the IκB/p65/COX-2/arachidonic acid pathway. 

At present, there are some limitations in this study, such as the metabolomics research only detected serum samples of mice, lacking the detection of colon tissue. And the potential bioactive components, key genes, biomarkers, and signaling pathways of JE for UC treatment that have not been validated by experimental pharmacology (Western blotting, RT‒PCR, and other in vitro or in vivo experiments). These limitations will be addressed in subsequent experimental pharmacology studies. Additionally, the sample size of this experiment was small, and only mouse models were used. Future studies may consider expanding the sample size, using clinical studies and conducting more experimental validation to explore the interaction between JE and other potential factors to more fully understand the pathogenesis of UC and the role of JE in UC.

## Conclusion

This study utilizes a sophisticated analytical approach that integrates network pharmacology and metabolomics to demonstrate, for the first time, that JE has the potential to reverse 2.5% DSS-induced UC in mice by modulating the IκB/p65/COX-2/arachidonic acid pathway. Our experimental findings not only offer a novel perspective on research ideas but also establish the groundwork for the clinical development of JE as a promising therapeutic agent for UC treatment.

### Supplementary Information

Supplementary Information.

## Data Availability

The original contributions presented in this experimental study are reflected in the articles/supplementary materials. Further inquiries can be directed to the corresponding authors.

## Abbreviations

AA: Arachidonic acid
ADME: Absorption, distribution, metabolism, and excretion
AUC: Area under curve
BSA: Bovine serum albumin
COX-2: Cyclooxygenase-2
DAB: Diaminobenzidine
DAI: Disease activity index
DL: Drug-likeness
DSS: Dextran Sulfate Sodium Salt
ELISA: Enzyme-linked immunosorbent assay
ESI: Electrospray ion source
FC: Fold change
H&E: Hematoxylin–Eosin
IBD: Inflammatory Bowel Disease
IHC: Immunohistochemistry
IL-10: Interleukin-10
IL-1β: Interleukin-1β
IL-6: Interleukin-6
IκB: Inhibitor of nuclear factor kappa-B
JE: *Jasminum elongatum* (Bergius) Willd.
KEGG: Kyoto Encyclopedia of Genes and Genomes
LPA: Lysophosphatidic acids
LPC: Lysophosphatidylcholine
LPI: Lysophosphatidylinositol
LSD: Least Significant Difference
NF-κB p65: Nuclear factor kappa-B p65
OB: Oral bioavailability
One-way ANOVA: One-way analysis of variance
OPLS-DA: Orthogonal partial least discriminant analysis
PBS: Phosphate buffer saline
PCA: Principal component analysis
PLS-DA: Partial least discriminant analysis
PPI: Protein–protein interaction
QC: Quality control
ROC: Receiver operator characteristic curve
SD: Standard deviation
TCM: Traditional Chinese Medicine
TNF-α: Tumor necrosis factor alpha
UC: Ulcerative colitis
UPLC-Q-TOF–MS: Ultra Performance Liquid Chromatography-Quadrupole-Time of Flight-Mass Spectrometry
VIP: Variable important in projection
WHO: World Health Organization
