# Supplementary material for: Integrated network pharmacology and metabolomics reveal the mechanisms of Jasminum elongatum in anti-ulcerative colitis
Source: Sci Rep. 2023 Dec 17;13:22449. doi: 10.1038/s41598-023-49792-w (PMC10725889; doi:10.1038/s41598-023-49792-w)
Supplement: Supplementary file 1 — Supplementary Information. [file 41598_2023_49792_MOESM1_ESM.docx]

**Supplementary Material**


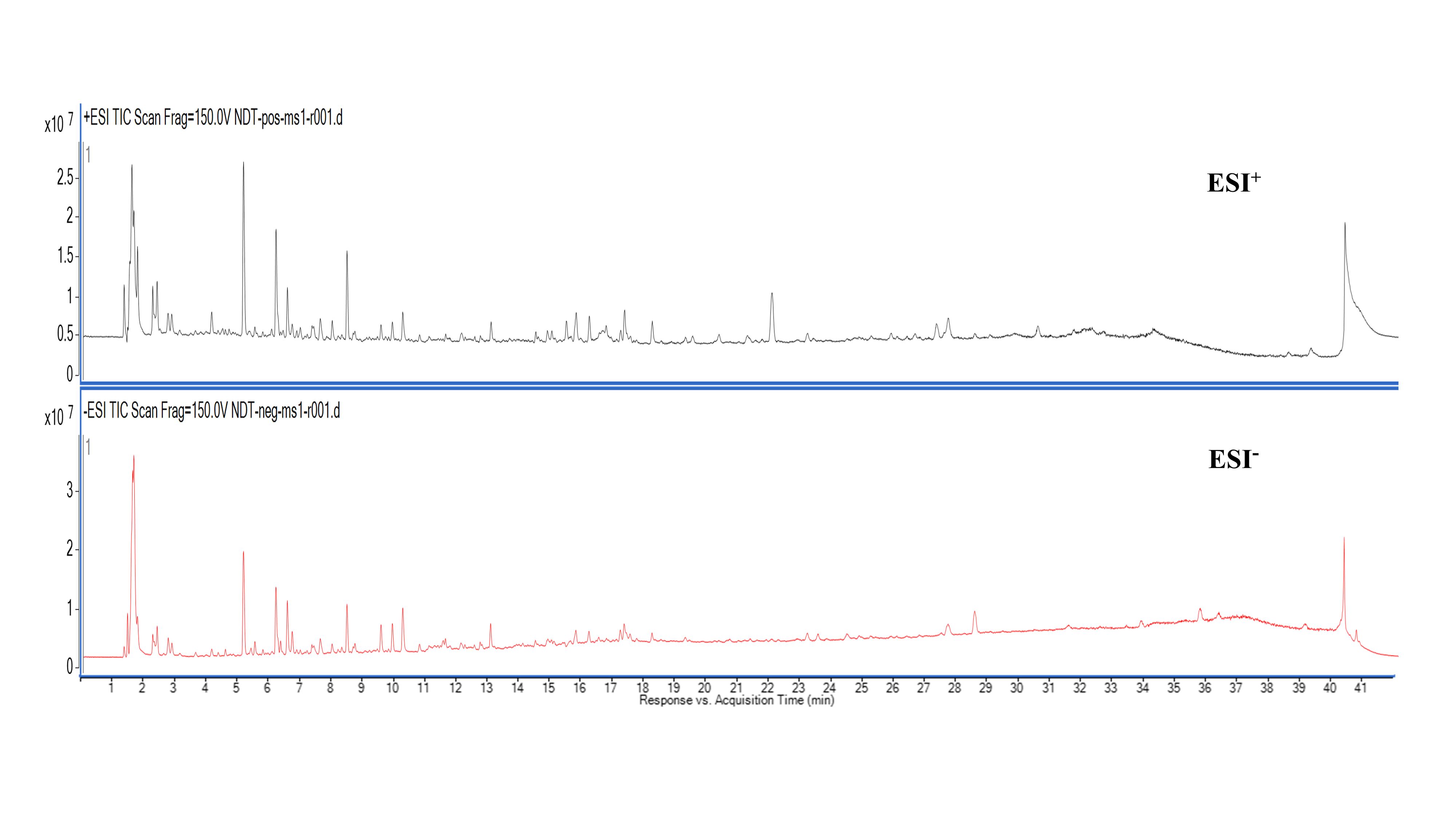


**Supplementary Fig.1** Total ion flow spectrum of JE.


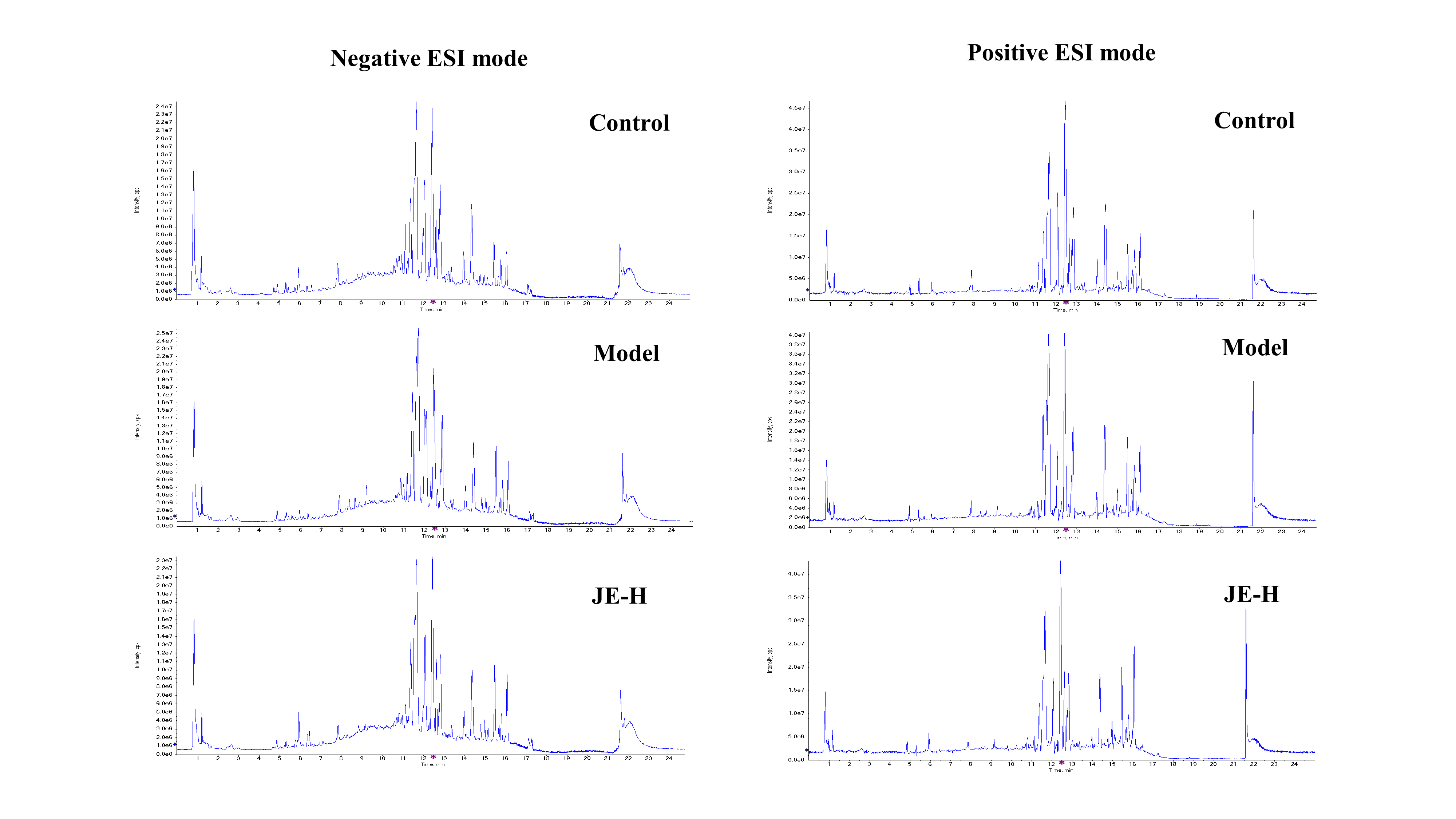


**Supplementary Fig.2.** Total serum ion flow diagram of mice in Control, Model and JE-H groups under negative and positive ion modes.
